# Her-2 Breast Cancer Outcomes Are Mitigated by Consuming n-3 Polyunsaturated, Saturated, and Monounsaturated Fatty Acids Compared to n-6 Polyunsaturated Fatty Acids

**DOI:** 10.3390/nu12123901

**Published:** 2020-12-20

**Authors:** Lyn M. Hillyer, Barbora Hucik, Enzo M. Baracuhy, Zhen Lin, William J. Muller, Lindsay E. Robinson, David W. L. Ma

**Affiliations:** 1 Department of Human Health and Nutritional Sciences, University of Guelph, Guelph, ON N1G 2W1, Canada; lhillyer@uoguelph.ca (L.M.H.); bhucik@uoguelph.ca (B.H.); ebaracuh@uoguelph.ca (E.M.B.); zlin01@mail.uoguelph.ca (Z.L.); 2 Department of Biochemistry, Rosalind and Morris Goodman Cancer Centre, McGill University, Montreal, QC H3A 1A3, Canada; william.muller@mcgill.ca

**Keywords:** fatty acid, breast cancer, n-3 PUFA, n-6 PUFA, MUFA, SFA

## Abstract

Lifestyle habits, such as the consumption of a healthy diet, may prevent up to 30–50% of breast cancer (BC) cases. Dietary fats are of specific interest, as research provides strong evidence regarding the association of dietary fats and BC. However, there is limited research on the role of different types of fats including polyunsaturated (PUFA), monounsaturated (MUFA), and saturated fatty acids (SFA). The objective of this study was to determine the effects of lifelong exposure to various dietary fats on mammary tumour development over a 20-week period. Female heterozygous MMTV-neu (ndl) YD5 mouse models were fed five maternal diets containing (1) 10% safflower oil (n-6 PUFA, control), (2) 3% menhaden oil + 7% safflower oil (marine n-3 PUFA, control), (3) 3% flaxseed + 7% safflower oil (plant-based n-3 PUFA), (4) 10% olive oil (MUFA), or (5) 10% lard (SFA). The primary measures, tumour latency, volume, and multiplicity differed by diet treatment in the following general order, n-6 PUFA > plant n-3 PUFA, SFA, MUFA > marine n-3 PUFA. Overall, these findings show that the quality of the diet plays a significant role influencing mammary tumour outcomes.

## 1. Introduction

Statistics show that 1 in 8 women will develop breast cancer (BC) during their lifetime [1]. A limited number of breast cancers, 5–10%, occur as a result of genetic predisposition alone [2]. Aside from non-modifiable risk factors, 30–50% of breast cancers can be prevented by lifestyle modifications such as consumption of healthy diets [3]. Epidemiological evidence supports the benefits of healthy diets in reducing BC risk. Specifically, in experimental and human studies, n-6 and n-3 polyunsaturated fatty acids (PUFA) have been extensively studied and shown to promote or attenuate BC outcomes, respectively [4,5,6,7,8,9,10].

Asian populations consuming diets rich in different n-3 PUFA, including marine-derived long-chain n-3 PUFA eicosapentaenoic acid (EPA, 20:5n-3) and docosahexaenoic acid (DHA, 22:6n-3), have a reduced incidence of BC [4]. However, Western populations with a high intake of n-6 PUFA, such as linoleic acid (LA, 18:2n-6), and n-3 PUFA intake lower than their Asian counterparts have a higher incidence of BC [7]. Asian-American women in the West have a 60% higher risk of BC [5] partially attributed to the high consumption of n-6 PUFA in the Western diet. N-6 PUFA, such as LA and arachidonic acid (AA, 20:4n-6), are thought to negatively affect health when present in large amounts in the diet [9]. Although not as potent as marine-derived n-3 PUFA, previous research [11] has shown that ALA from flaxseed oil also possesses protective effects in BC in a dose-dependent manner. It is apparent that balance is important as consuming significant quantities of either n-6 or n-3 PUFA results in different outcomes. For example, an in vitro study demonstrated that higher ratios of AA to EPA and DHA resulted in differential growth of MCF-7 BC cells [12]. Similarly, a case-control study found both eliminating fish from one’s diet combined with consuming high n-6:n-3 ratios independently increased the risk of developing BC [13].

Mediterranean countries have reduced rates of cardiovascular diseases and cancers attributed to Mediterranean dietary patterns rich in fish, nuts, fruit and vegetables, and olive oil [14,15,16]. Therefore, monounsaturated fatty acids (MUFA)—such as oleic acid (18:1n9, OA), which are abundant in olive oil—are of particular interest when studying the potential beneficial effects of dietary fat. A meta-analysis of postmenopausal BC showed that adherence to the Mediterranean diet was significantly associated with a decrease risk in estrogen receptor negative (ER-) BC [17].

Saturated fatty acids (SFA) have been extensively studied in relation to cardiovascular health, but less is known regarding the specific effects of SFA consumption on BC. A few studies have shown a weak association between SFA consumption and BC risk [8,18,19]. However, a recent case-cohort analysis found that BC risk was elevated in women with a greater percent incorporation of palmitic acid (16:0, PA) into plasma phospholipids [20]. However, this may not be a direct reflection of intake as SFA can be endogenously synthesized from acetyl CoA when consuming a high carbohydrate diet [21]. A 2015 meta-analysis found a positive association between high SFA intake and BC incidence in postmenopausal women from case-control studies but not cohort studies [22]. 

Based on current evidence, it is evident that different families of fatty acids may promote or protect against BC. What remains to be studied, however, is a direct comparison between families of fatty acids to better understand their relative impact on BC outcomes. Thus, the objective of this study was to directly compare the effects of different families of dietary fatty acids, and specifically how they influence mammary tumour latency, volume, and multiplicity in a mouse model of Her-2 breast cancer (MMTV-neu(ndl)-YD5). Since Her-2 BC is prevalent in 10–25% of women [23], the MMTV-neu(ndl)-YD5 mouse is a relevant model for exploring prevention and treatment strategies. It was hypothesized that mice fed n-3 PUFA and MUFA would have longer latency, fewer and smaller tumours compared to n-6 PUFA fed mice. Given the unhealthy associations with SFA, it was hypothesized that mice fed SFA would have the poorest outcomes relative to all groups. This study contributes to the comprehensive understanding of how diets rich in different families of fatty acids contribute to BC risk.

## 2. Materials and Methods 

### 2.1. Animals and Diets

Each harem consisted of one male heterozygous MMTV-neu(ndl)-YD5 mouse and three female FVB wild type mice. Harems were randomly assigned to one of five diets (Research Diets, Inc., New Brunswick, NJ, USA; Table 1): (1) 10% w/w safflower oil (n-6 PUFA control; *n* = 11), (2) 3% *w*/*w* menhaden oil + 7% w/w safflower oil (marine n-3 PUFA control; *n* = 10), (3) 3% w/w flaxseed oil + 7% w/w safflower oil (plant-based n-3 PUFA *n* = 9), (4) 10% w/w olive oil (MUFA; *n* = 6) and (5) 10% w/w lard diets (SFA; *n* = 10). These are isocaloric and physiologically relevant diets based on our previous work [11]. Mice were provided ad libitum access to diet and double-distilled water. Food intake was recorded 3 times per week. Fatty acid composition of diets was confirmed by gas chromatography (Table 1). Macronutrient composition of diets was provided by the manufacturer (Appendix A). All experimental procedures were approved by the University of Guelph’s Animal Care Committee (#3488) in 2015. Offspring were weaned and genotyped at three weeks of age as described previously [24]. Female transgenic offspring were placed on maternal diets and male offspring were terminated. Mice were housed together in ventilated cages with a maximum number of 4 mice per cage.

### 2.2. Mammary Tumour Measurements and Tissue Collection

Starting at 10 weeks of age, tumours were palpated and measured using a digital caliper. Tumour measurements were taken 3 times per week when a new tumour was detected until termination at 20 weeks of age. Tumour volume was calculated using V = [length × (width^2^)]/2. Mice were terminated at 20 weeks of age by CO_2_ overdose. If tumour dimensions exceeded 17 mm in length or width, or had tumours more than 5000 mm^3^, mice were terminated prior to the 20-week endpoint. Mice were terminated if in proestrus, estrus, or metestrus stages of the estrous cycle. If in the diestrus stage, termination was delayed for a maximum of 2 days to control for hormonal fluctuations. To determine stage of estrous cycle, the vagina was flushed with 30 μL of phosphate buffer solution. The solution was observed under a microscope (Nikon Eclipse TS100, Neville Instruments, Melville, NY, USA) on a glass slide. At time of termination, blood was collected by cardiac puncture. After 20 min of clot time, blood was spun down at 357× *g* for 5 min) to separate red blood cells from plasma. The mouse pelt with mammary gland and tumours attached was removed for measurements of final tumour dimensions, then tumours and mammary glands were removed, weighed, and stored at −80 °C for future analysis.

### 2.3. Fatty Lipid Analysis

Lipid composition of tumours was determined by gas chromatography (GC) as described previously [25]. Fatty acid composition was expressed as a percentage of total fatty acids. In brief, lipids were extracted by chloroform:methanol and transmelthylated with boron trifluoride. They were separated and analyzed by GC (7890A Agilent Technologies, SpectraLab, Markham, ON, Canada).

### 2.4. Statistical Analysis

SAS version 9.1 (SAS Institute, Cary, NC, USA) was used for all statistical analyses. The upper limit of probability for statistical significance was set at *p* ≤ 0.05. A one-way ANOVA was conducted to detect differences in tumour latency and fatty acid composition of tumours followed by Tukey’s Studentized Range test. A repeated measures analysis was applied to tumour volume and multiplicity to detect differences between diets over the 20-week experimental period.

## 3. Results

### 3.1. Tumour Latency and Tumour-Free Status

**Figure 1:**
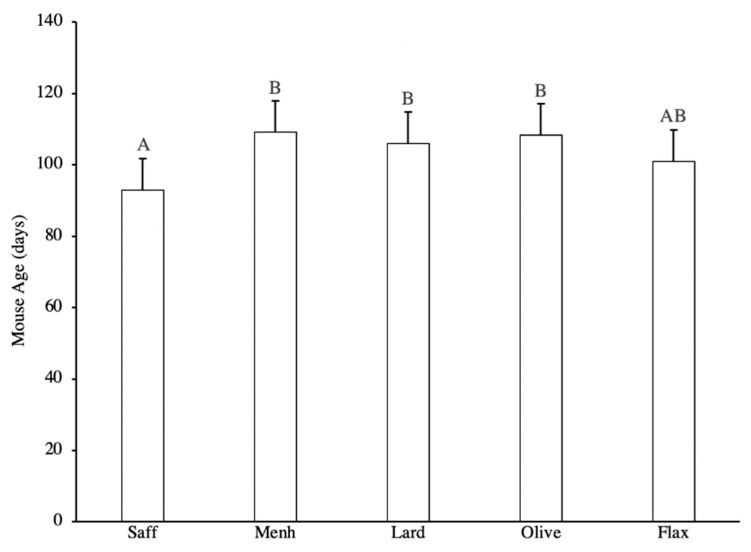
Average tumour latency in mice fed either 10% safflower oil diet (*n* = 11), 3% menhaden oil diet (*n* = 10), 3% flaxseed diet (*n* = 9), 10% olive oil diet (*n* = 6), or 10% lard diet (*n* = 10). Groups with a different letter are significantly different according to Tukey’s Studentized Range test (*p* < 0.05).

**Figure 2:**
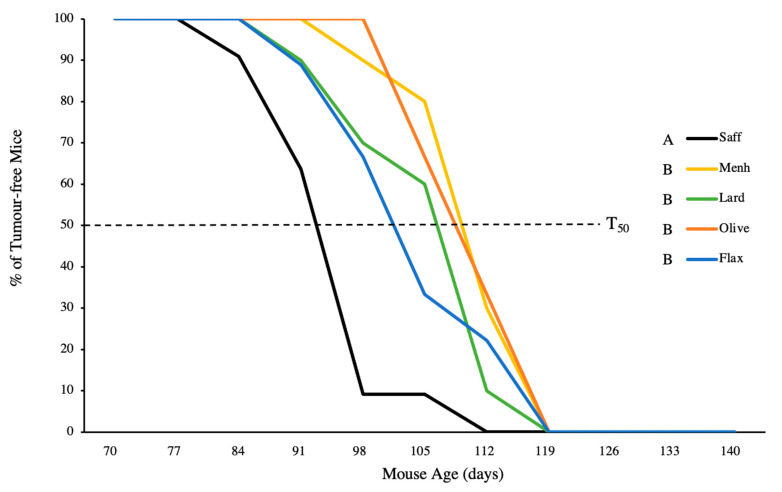
Proportion of mice that remained tumour free over the 20-week experimental period fed either 10% safflower oil diet (*n* = 11), 3% menhaden oil diet (*n* = 10), 3% flaxseed diet (*n* = 9), 10% olive oil diet (*n* = 6), or 10% lard diet (*n* = 10). T50 denotes the time at which 50% of mice in a given group developed tumours. Groups with a different letter are significantly different as determined by repeated measures analysis (*p* < 0.05).

Tumour onset was significantly earlier in safflower-fed mice enriched in n-6 PUFA (Figure 1). Average tumour latency was extended in mice fed olive oil, menhaden, and lard diets compared to the safflower n-6 PUFA control, while flaxseed was intermediate (Figure 1). Safflower-fed mice reached their T50, the median age when 50% of mice developed tumours, significantly faster than other diets and no significant differences in T50 were observed between the other diets (Figure 2).

### 3.2. Tumour Volume

**Figure 3:**
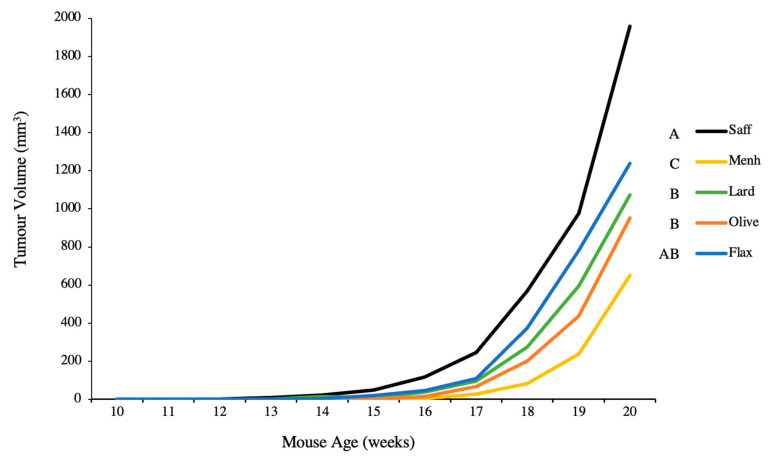
Average tumour volume per dietary intervention over the duration of the study in mice fed either 10% safflower oil diet (*n* = 11), 3% menhaden oil diet (*n* = 10), 3% flaxseed diet (*n* = 9), 10% olive oil diet (*n* = 6), or 10% lard diet (*n* = 10). Groups with a different letter are significantly different as determined by repeated measures analysis (*p* < 0.05).

The menhaden n-3 PUFA control significantly reduced tumour volume compared to all other diets, and the safflower n-6 PUFA control significantly increased tumour volume compared to all other diets except flaxseed (Figure 3). No significant differences in tumour volume were found among the flaxseed oil, olive oil, and lard diets (Figure 3).

### 3.3. Tumour Multiplicity

**Figure 4:**
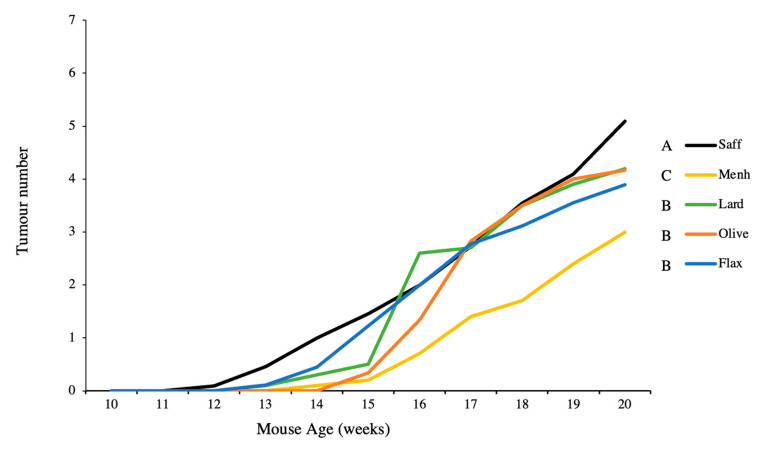
Average tumour number per dietary intervention over the duration of the study in mice fed either 10% safflower oil diet (*n* = 11), 3% menhaden oil diet (*n* = 10), 3% flaxseed diet (*n* = 9), 10% olive oil diet (*n* = 6), or 10% lard diet (*n* = 10). Groups with a different letter are significantly different as determined by repeated measures analysis (*p* < 0.05).

The safflower n-6 PUFA control significantly increased tumour multiplicity compared to all other diets, and the menhaden control significantly decreased tumour multiplicity compared to all other diets (Figure 4). No significant differences in tumour multiplicity were found among the flaxseed oil, olive oil, and lard diets (Figure 4).

### 3.4. Fatty Acid Composition of Tumour Phospholipids

Phospholipid analysis of mammary tumours was conducted to determine if the observed effects on primary tumour outcomes could be attributed to the incorporation of specific dietary fatty acids into the target tissue. The experimental diets enriched in different families of fatty acids were found to be also enriched in the phospholipid fractions (phosphatidylcholine, PC; phosphatidylethanolamine, PE) in mammary tumours. The percent composition is reported in Table 2 and Table 3.

In the PC fraction (Table 2), the mammary tumours of mice fed olive oil or lard had higher amounts of OA (18:1c9) compared to menhaden, safflower and flaxseed oil-fed mice, although olive oil had higher OA amounts than lard. Mice fed flaxseed had the highest amount of LA (18:2n-6) present in tumours, safflower, and menhaden had less than flaxseed, and lard and olive oil had the least amount of LA (18:2n-6) out of all the fatty acids. EPA (20:5n-3) was increased in mice fed menhaden compared to olive oil, and DHA (22:6n-3) was increased in mice fed menhaden oil compared to all diets except for flaxseed. Flaxseed had higher levels of ALA compared to all other diets in the PC fraction.

In the PE fraction (Table 3), mammary tumours of mice fed lard had higher amounts of OA compared to mice fed safflower or menhaden. The mammary tumours of mice fed olive oil were composed of higher quantities of OA (18:1c9) compared to all diets except for lard. The levels of LA (18:2n-6) present in tumours were as follows, flax > menhaden, safflower > lard > olive oil. n-6 DPA was significantly increased in mammary tumours of mice fed safflower oil compared to all other diets. DHA (22:6n-3) was increased in mammary tumours of mice fed menhaden oil compared to all other diets, and safflower had the least amount. 

## 4. Discussion

Using the MMTV-neu (ndl)YD5 transgenic mouse model, the present study compared the effects of different diets enriched in either n-6 PUFA, n-3 PUFA (marine and plant), MUFA, or SFA on BC outcomes in the same animal study. A critical limitation of past studies is the relatively limited comparisons typically between n-3 and n-6 PUFA, which has led to the conclusion that one type of PUFA inhibits and the other promotes cancer, a potential over-simplification that needs to be acknowledged. In the current work, including a broad range of all major families of fatty acids in the same animal study showed that mice fed SFA did not have the poorest outcomes as originally hypothesized. Rather, SFA exerted similar effects to MUFA and plant-derived n-3 PUFA with respect to tumour outcomes. Importantly, findings from this study, which includes a more balanced inclusion of all major families of fatty acids, support the long-standing view that n-6 PUFA promotes while marine-derived n-3 PUFA mitigates tumour outcomes.

### 4.1. Role of Dietary Fat in Modulating Mammary Tumour Outcomes

Experimental findings show that a diet high in both ALA and LC n-3 PUFA attenuates tumorigenesis in BC, inhibiting BC growth, angiogenesis and metastasis, compared to diets high in n-6 PUFA [11,26,27,28,29,30,31]. Although epidemiological evidence shows an association between increased dietary n-3 PUFA content and decreased BC occurrence in humans [4,6,7], more conclusive findings have been found in animal studies, especially regarding the dose-dependent effects by which both plant- and marine-derived n-3 PUFA exert anti-tumourigenic properties [11,25]. A comparison of marine-derived versus plant-derived n-3 PUFA [23] found that marine-derived n-3 PUFA (EPA and DHA) were more effective at mitigating BC outcomes compared to the same w/w dose of plant-derived n-3 PUFA (ALA). Interestingly, increasing the dose of plant-derived n-3 PUFA led to similar antitumourigenic effects as the original w/w content of marine-derived n-3 PUFA, indicating that marine-derived n-3 PUFA are more powerful than plant-derived n-3 PUFA on a w/w basis [23]. The present study extends this body of work by demonstrating that relative to all other major families of fatty acids, marine-derived n-3 PUFA, EPA, and DHA, are clearly the most potent anti-tumourigenic fatty acids in this BC model.

According to our findings, olive oil was not as effective as marine-derived n-3 PUFA at reducing BC outcomes at physiologically-relevant quantities, though it was better than n-6 PUFA. More research into its dose-dependent effects are needed. In humans, the Primary Prevention of Cardiovascular Disease with a Mediterranean Diet (PREDIMED) study found a 62% reduction in BC risk in participants supplementing their diet with extra-virgin olive oil compared to the control group [14]. Furthermore, OA, a MUFA found in olive oil, was found to inhibit tumour cell growth in a MCF-7 BC cell line [32], and also had anti-proliferative effects in vitro through the modulation of cancer-promoting oncogenes [15]. A human cohort study of over 23,000 participants cataloging their food intake 7 days/week over a 16-year period suggests OA intake lowered the incidence of pancreatic cancer [33]. Contrarily, more recent evidence suggests the anti-cancer properties of olive oil might be attributed to the totality of other compounds, such as phenolic acids present in the olive oil rather than the OA itself [16,34,35]. In vitro studies of prostate cancer [36] and gastric cancer [37] cells found that OA had pro-proliferative effects. Pro-tumourigenic effects of OA in mice with cervical cancer have also been described [38]. One study considered these discrepancies and analyzed the effects of olive oil as well its individual constituents on colorectal cancer cell cultures [35]. In this study, cells treated with only OA compared to OA and other components of olive oil have opposite effects on the proliferation of colorectal cells [35], suggesting that anti-cancer effects of olive oil are attributed to other compounds in olive oil rather than OA. Finally, an in vitro study found a negative correlation between quantity of these other compounds and the proliferation of bladder cancer cells [39]. Further research is required to assess the specific anticancer effects attributable to OA and phenolic compounds present in olive oil, particularly in in vivo models. 

Consistent with this growing body of literature, the present study demonstrated that SFA had an intermediate effect on tumour outcomes and were not strongly pro-tumourigenic in this BC model. Although traditionally viewed as having negative health effects, SFA may not necessarily exert pro-tumourigenic effects as there is only a weak association between SFA consumption and BC risk [8,18,19]. The association between SFA consumption and BC risk may be dependent on age and duration of exposure, as one study found that younger women had an increased risk of BC associated with SFA consumption, while older women had a reduced risk [20]. Though age was not a factor in the present study, these results suggest the need to consider the age of subjects when conducting diet-based studies. Research examining the effects of specific SFA found that lauric acid, a 12-carbon SFA stimulated normal mammary gland development in vivo [40], while stearic acid, an 18-carbon SFA, suppressed mammary gland development both in vivo and in vitro through the inhibition of the PI3K/Akt signaling pathway [40]. Additionally, exposure to high levels of lard in utero decreased BC risk later in life, an effect attributed to dietary modulation of mammary gland development [41]. An in vitro study found that palmitic acidhad proliferative effects on pancreatic cells through the TLR4/ROS/NF-κB/MMP-9 signaling pathway [42], though they did not directly compare these effects to other fatty acids. A cell-line study comparing similar groups of fatty acids as the present study on normal and cancerous cells showed that stearic acid consistently resulted in lower cell numbers among all the fatty acids in both cancerous and normal cell lines [43]. These in vitro studies suggest that the length of SFA might be a factor influencing cancer outcomes. Observations also vary based on the source of SFA, as dairy consumption exerts different effects compared to red meat consumption. Total dairy consumption, not including milk, is associated with a reduced risk of BC [44]; however, increased intake of red meat is associated with an increased risk of BC [45].

### 4.2. Tumour Fatty Acid Composition and Tumourigenesis

Evidence suggests that membrane fatty acid composition of tissues can affect cellular signaling through the modification of lipid rafts. Lipid rafts in the cell membrane of a cell act as microdomains to facilitate signaling events [46], particularly those involved in pro-tumourigenic activities promoting tumour cell proliferation and survival [6,47]. Although we did not analyze lipid rafts directly, indirect evidence is provided by examining the phospholipid fatty acid composition of PC and PE in mammary tumours. Significant increases in OA in PC and PE fractions were observed in mice fed an olive oil or lard diet compared to safflower, menhaden, and flax diets. OA was also significantly increased in the PC and PE fractions in mice fed the lard diet compared to all other diets. This was surprising and may be due to the fact that SFA can be converted to OA through elongation and desaturation of palmitic and stearic acid precursors found in lard [48]. This evidence suggests that SFA and MUFA may exert their biological effects through cell membrane mediated mechanisms. 

Analysis of mammary tumours showed significant increases in n-3 PUFA, specifically DHA, in the PC and PE fractions in mice fed a menhaden oil diet, as well as DPA in the PE fraction, suggesting that EPA may be incorporated initially and further elongated. As the menhaden oil diet contained 7% safflower oil, which is rich in n-6 PUFA, the presence of DPA and DHA in tumour tissue indicates that n-3 PUFA are preferentially used as a substrate. This notion is important in eicosanoid synthesis, as eicosanoids derived from AA are thought to promote inflammation and subsequent tumourigenesis but have protective effects when derived from EPA and DHA [49,50,51]. Inflammation and eicosanoid synthesis from n-6 PUFA could explain the poor tumour outcomes observed in the safflower diet, which had higher levels of most n-6 PUFA compared to other diets. It is important to note that emerging views about AA have shown that different downstream metabolites of AA to be both anti-inflammatory and pro-inflammatory, which suggests a complex balance of various metabolites may be involved in tumour outcomes [52,53]. n-3 PUFA affect lipid raft composition and may inhibit pro-tumourigenic signaling pathways engendered from highly organized lipid rafts. Incorporation of n-3 PUFA negatively alters the organization of lipid rafts and potentially lipid raft-modulated signaling compared to SFA. One study found that DHA treatment modulated levels of OA in lipid rafts of T cells, therefore n-3 PUFA can modulate SFA and MUFA composition of lipid rafts although the mechanism is unclear [54]. Specifically, in HER2+ BC, DHA reduced HER-2 signaling through the disruption of lipid rafts. The modulation of lipid rafts by n-3 PUFA could potentially be used as an anti-tumourigenic therapy to disrupt cancer-promoting signaling pathways [55]. DHA particularly has an effect on downregulating the expression of onco-proteins through modulating apoptosis, which is inhibited in cancer, therefore promoting tumour cell survival [48]. Therefore, inhibiting signaling cascades that promote tumour growth may reduce tumour size and number. This property of n-3 PUFA could explain the better tumour outcomes observed in the menhaden diet compared to olive oil, flax, and lard, which all had lower levels of EPA and DHA compared to menhaden.

### 4.3. Relevance of Model and Diets 

The mouse mammary tumour virus MMTV-neu(ndl)-YD5 model was developed to provide a murine equivalent to study HER2+ BC in humans. Due to its highly aggressive phenotype, this model develops mammary tumours by 100 days of age, providing a relevant model to research dietary strategies to modulate tumourigenesis in BC [56]. 

All diets in the present study provided mice with 22% of total caloric intake from fat, which is physiologically reflective of a moderate fat intake in humans typically consuming ~30% of daily energy from fat [57]. Relative to dietary fat composition, the experimental diets consumed by the mice were also close approximations. On average, mice typically consume 2 g of food per day [11]. For mice consuming the diet containing menhaden oil, this quantity translates to 1.6% of total daily energy from EPA and DHA, while a traditional Japanese diet provides 1–2% of daily energy from EPA and DHA [7]. For safflower oil, mice consumed 16% of their daily energy from LA, which is modestly higher than the average American intake of 5–6% and 5–10% recommended by the Institute of Medicine [58]. However, observational studies of humans consuming up to 21% over 11 years have been noted to have no adverse effects [58]. For the flaxseed oil treatment, mice consumed 3.8% of their daily energy as ALA, which is higher than the average American consuming 0.2–0.7% (0.7–1.6 g/day) of total calories per day as n-3 PUFA, most of which is ALA [59]. The lard diet provided 8% of the daily caloric intake from saturated fatty acids, which approximates the 2015 US Dietary Guidelines Advisory Committee’s recommendation of a 10% maximum daily intake [60]. The olive oil diet delivered 14% of daily caloric intake from MUFA, and while there is no formal recommended intake for MUFA, a French opinion suggests MUFA to encompass 15–20% of total daily energy intake [61].

## 5. Strengths, Limitations, and Future Directions

A major strength of this work is the evaluation of major families of fatty acids together in one study, which provides a more complete picture of how fatty acids influence BC development. This is an important study to direct future work, but some limitations are acknowledged that can be addressed in future studies. There are many different forms of BC, therefore examining effects in hormone dependent and hereditary types of cancers is needed. Further work investigating dose-dependent effects of MUFA and SFA is required as this study only tested one level. It is important to consider that SFA and MUFAs can be produced endogenously [48], thus the outcomes of the diets could have been influenced by fatty acids synthesized de novo. To prevent such confounding, future studies using the fatty acid synthase (FASN) and stearoyl-CoA desaturase 1 (SCD1) knockout models would be relevant [62,63]. In addition to the type of fat, the total amount of fat needs to be considered. This study employed a moderate amount of dietary fat; comparison of low and high fat diets is also warranted given that obesity is risk factor for BC. The adipose tissue surrounding tumours is also known to influence tumour progression; thus, examination of lipid droplets around tumour tissues could provide more insight into factors contributing to the tumour phenotype. Future studies should also explore the potential of dietary fats to mitigate metastasis of tumours. Finally, future studies examining mechanisms of action—such as inflammatory, cell cycle control, oncogene, and protein expression—are needed to understand the fundamental basis by which individual fatty acids mediate their actions.

## 6. Conclusions

For simplicity, dietary studies often are a comparison of two different diets, which gives rise to a ‘good vs. bad’ dietary conclusion. By comparing all the major families of fatty acids together using enriched oils, this study was able to provide a relativistic view of their influence on BC outcomes. In conclusion, marine-derived n-3 PUFA best mitigates BC outcomes compared to other dietary fatty acid examined in this study. MUFA, SFA, and plant-derived n-3 PUFA performed similarly and n-6 PUFA fed mice had the poorest BC outcomes. Overall, these findings highlight the need to carefully consider the type of fatty acid in dietary lifestyle interventions for the prevention of BC.

## Figures and Tables

**Table 1:** Fatty acid composition of diets.

Fatty Acid	10% Safflower Oil	3% Menhaden Oil	3% Flaxseed Oil	10% Olive Oil	10% Lard
12:0	0.04	0.08	0.03	0.04	1.49
14:0	0.24	2.67	0.22	0.16	1.28
15:0	0	0.26	0.03	0	0.11
16:0	6.91	10.11	6.52	14.51	21.27
16:1c9	0.15	3.99	1.13	1.98	1.67
17:1c10	0	0.19	0.02	0.13	0.24
18:0	2.59	2.85	2.89	2.11	11.51
18:1c9	15.25	13.78	16.59	64.50	35.76
18:1c11	0.80	1.47	0.78	3.51	2.33
18:2n6	71.93	51.71	53.81	11.15	20.85
18:3n6	0.11	0.16	0	0	0.11
19:0	0	0	0	0	0.10
19:1c7	0	0.18	0	0	0
18:3n3	0.31	0.86	17.30	0.66	1.56
18:4n3	0.13	0.90	0.14	0	0.17
20:0	0.36	0.36	0.51	0.39	0.22
20:1c5&8	0.10	0	0	0	0
20:1c11	0.22	0.49	0.34	0.37	0.79
20:2n6	0.05	0.12	0.09	0	0.84
20:3n6	0	0.10	0	0	0.17
20:4n6	0	0.49	0	0	0.37
20:3n3	0	0.20	0	0	0.25
20:5n3	0.05	4.20	0	0	0
22:0	0.30	0.27	0.28	0.13	0
22:1n9	0.02	0.09	0.02	0.03	0.02
22:2n6	0	0.27	0	0	0
22:4n6	0.11	0.14	0	0	0.12
22:5n3	0	0.75	0	0	0.12
24:0	0.14	0.02	0.16	0.12	0.01
22:6n3	0	3.08	0	0	0
24:1	0.17	0.23	0.15	0.19	0
Total n-6	72.20	52.99	53.90	11.15	22.46
Total n-3	0.49	9.99	17.44	0.66	2.1
Total SFA	10.58	16.62	10.64	17.46	35.99
Total MUFA	16.71	43.19	19.03	70.71	40.81
Total PUFA	72.69	62.98	71.34	11.81	24.56

Fatty acid composition (%) of safflower, flaxseed, menhaden, olive and lard diets. Lipids were extracted and analyzed from 3 separate pellets per diet and analyzed by gas chromatography. Averages are shown.

**Table 2:** Percent composition of fatty acids in PC fraction of mammary tumour phospholipids.

Fatty Acids	10% Safflower	3% Menhaden	3% Flaxseed	10% Olive	10% Lard
12:0	0.04 ± 0.06	0.15 ± 0.09	0.09 ± 0.01	0.30 ± 0.29	0.07 ± 0.03
14:0	1.06 ± 0.31	1.25 ± 0.34	0.81 ± 0.06	0.83 ± 0.33	1.25 ± 0.16
15:0	0.24 ± 0.04	0.24 ± 0.17	0.22 ± 0.03	0.24 ± 0.05	0.25 ± 0.02
16:0	29.21 ± 1.16	29.39 ± 1.01	27.75 ± 2.08	25.81 ± 2.77	27.74 ± 3.07
18:0	8.32 ± 1.04	8.61 ± 2.58	8.24 ± 0.82	6.95 ± 1.68	7.25 ± 1.21
19:0	0.13 ± 0.08	0.14 ± 0.07	0.13 ± 0.02	0.15 ± 0.08	0.10 ± 0.03
20:0	0.11 ± 0.03	0.12 ± 0.05	0.19 ± 0.18	0.08 ± 0.02	0.08 ± 0.01
24:0	0.33 ± 0.13 ^B^	0.56 ± 0.23 ^A^	0.24 ± 0.05 ^B^	0.24 ± 0.06 ^B^	0.14 ± 0.07 ^B^
Total SFA	39.44	40.47	37.68	34.60	36.88
16:1c-9	2.80 ± 0.79	3.34 ± 1.01	2.84 ± 0.33	3.62 ± 1.06	3.84 ± 0.44
18:1c-9	13.03 ± 1.60 ^C^	13.76 ± 1.72 ^C^	13.80 ± 1.61 ^C^	23.63 ± 1.23^A^	19.88 ± 1.10 ^B^
18:1c-11	5.16 ± 0.61 ^B^	6.92 ± 1.94 ^AB^	5.37 ± 0.92 ^B^	7.86 ± 1.03 ^A^	8.14 ± 1.28 ^A^
20:1c-11	0.86 ± 0.31	0.83 ± 0.41	0.74 ± 0.33	1.28 ± 0.27	1.11 ± 0.20
22:1n-9	0.48 ± 0.14	0.37 ± 0.17	0.18 ± 0.09	0.37 ± 0.18	0.34 ± 0.24
24:1n-9	0.52 ± 0.25	0.59 ± 0.09	0.26 ± 0.05	0.57 ± 0.38	0.41 ± 0.08
Total MUFA	22.85	25.80	23.19	37.33	33.71
18:2n-6	13.11 ± 1.70 ^B^	12.34 ± 1.83 ^B^	17.33 ± 2.16 ^A^	4.79 ± 0.37 ^C^	7.20 ± 0.58 ^C^
18:3n-6	0.22 ± 0.09 ^A^	0.11 ± 0.05 ^B^	0.17 ± 0.01 ^AB^	0.13 ± 0.03 ^AB^	0.10 ± 0.02 ^B^
18:3n-3	0.21 ± 0.22 ^B^	0.21 ± 0.08 ^B^	0.49 ± 0.19 ^A^	0.19 ± 0.08 ^B^	0.18 ± 0.05 ^B^
20:2n-6	2.24 ± 0.75 ^A^	1.77 ± 0.65 ^AB^	1.98 ± 0.61 ^A^	0.45 ± 0.11 ^C^	1.02 ± 0.17 ^BC^
20:3n-6	3.40 ± 1.43 ^BC^	2.92 ± 0.51 ^BC^	4.18 ± 0.67 ^B^	2.21 ± 0.36 ^C^	2.47 ± 0.27 ^C^
20:4n-6	13.89 ± 3.43 ^AB^	8.85 ± 2.13 ^C^	10.68 ± 1.93 ^BC^	16.81 ± 2.50 ^A^	15.03 ± 1.66 ^AB^
20:5n-3	0.54 ± 0.73 ^BC^	0.89 ± 0.31 ^B^	0.50 ± 0.16 ^BC^	0.16 ± 0.05 ^C^	0.19 ± 0.04 ^BC^
22:2n-6	0.31 ± 0.10 ^A^	0.22 ± 0.06 ^AB^	0.20 ± 0.07 ^AB^	0.17 ± 0.09 ^AB^	0.11 ± 0.07 ^B^
22:4n-6	1.20 ± 0.16 ^A^	0.51 ± 0.16 ^B^	0.39 ± 0.06 ^B^	0.74 ± 0.36 ^B^	0.61 ± 0.06 ^B^
22:5n-6	1.10 ± 0.34 ^A^	0.47 ± 0.19 ^B^	0.18 ± 0.04 ^B^	0.46 ± 0.23 ^B^	0.38 ± 0.09 ^B^
22:5n-3	0.41 ± 0.31 ^B^	1.49 ± 0.61 ^A^	0.83 ± 0.03 ^B^	0.36 ± 0.15 ^B^	0.36 ± 0.10 ^B^
22:6n-3	1.06 ± 0.50 ^B^	3.95 ± 2.89 ^A^	2.19 ± 0.29 ^AB^	1.62 ± 0.22 ^B^	1.77 ± 0.11 ^B^
Total PUFA	37.70	33.73	39.13	28.07	29.41

Within a row, values not sharing a letter are significantly different according to a one-way ANOVA (*p* < 0.05). Values are expressed as mean ± standard deviation (*n* = 6–11).

**Table 3:** Percent composition of fatty acids in PE fraction of mammary tumour phospholipids.

Fatty Acids	10% Safflower	3% Menhaden	3% Flaxseed	10% Olive	10% Lard
12:0	0.04 ± 0.10 ^B^	0.05 ± 0.08 ^B^	0.13 ± 0.07 ^AB^	0.26 ± 0.07 ^A^	0.23 ± 0.12 ^A^
14:0	0.40 ± 0.12	0.42 ± 0.19	0.39 ± 0.07	0.33 ± 0.14	0.53 ± 0.16
15:0	0.18 ± 0.14	0.09 ± 0.11	0.15 ± 0.09	0.20 ± 0.13	0.24 ± 0.11
16:0	6.79 ± 0.74 ^A^	7.48 ± 0.65 ^A^	7.22 ± 0.66 ^A^	5.00 ± 0.50 ^B^	6.70 ± 0.25 ^A^
18:0	14.25 ± 1.28	13.93 ± 2.46	13.70 ± 1.12	12.03 ± 1.95	11.84 ± 1.32
19:0	0.14 ± 0.13	0.04 ± 0.07	0.12 ± 0.09	0.04 ± 0.10	0.08 ± 0.10
20:0	0.15 ± 0.03	0.11 ± 0.04	0.10 ± 0.02	0.14 ± 0.05	0.09 ± 0.09
24:0	0.79 ± 0.29 ^A^	0.63 ± 0.28 ^AB^	0.36 ± 0.19 ^B^	0.54 ± 0.13 ^AB^	0.33 ± 0.22 ^B^
Total SFA	22.75	22.75	22.18	18.54	20.04
16:1c9	1.45 ± 0.34 ^B^	1.90 ± 0.37 ^A^	1.90 ± 0.22 ^A^	1.27 ± 0.28 ^B^	1.92 ± 0.17 ^A^
18:1c9	17.55 ± 2.61 ^C^	18.20 ± 4.59 ^C^	19.96 ± 1.32 ^BC^	25.04 ± 3.17 ^A^	24.08 ± 0.73 ^AB^
18:1c11	4.77 ± 0.96 ^B^	5.65 ± 1.48 ^AB^	5.38 ± 0.89 ^AB^	5.83 ± 1.23 ^AB^	6.83 ± 1.22 ^A^
20:1c11	1.07 ± 0.34 ^AB^	0.88 ± 0.45 ^AB^	0.74 ± 0.31 ^B^	1.34 ± 0.30 ^A^	1.23 ± 0.28 ^AB^
22:1n-9	1.36 ± 1.37 ^A^	0.28 ± 0.09 ^AB^	0.06 ± 0.10 ^B^	0.34 ± 0.11 ^AB^	0.21 ± 0.02 ^B^
24:1n-9	0.23 ± 0.08 ^AB^	0.10 ± 0.12 ^B^	0.17 ± 0.06 ^AB^	0.32 ± 0.10 ^A^	0.15 ± 0.07 ^B^
Total MUFA	26.44	27.02	28.21	34.14	34.41
18:2n-6	7.52 ± 0.80 ^B^	8.75 ± 1.23 ^B^	11.10 ± 1.98 ^A^	2.41 ± 0.47 ^D^	4.38 ± 0.37 ^C^
18:3n-6	0.19 ± 0.16 ^A^	0.05 ± 0.06 ^AB^	0.12 ± 0.11 ^AB^	0.01 ± 0.03 ^B^	0.04 ± 0.04 ^AB^
18:3n-3	0.22 ± 0.34 ^AB^	0.02 ± 0.05 ^B^	0.46 ± 0.26 ^A^	0.13 ± 0.14 ^AB^	0.23 ± 0.12 ^AB^
20:2n-6	1.72 ± 0.55 ^A^	1.14 ± 0.30 ^ABC^	1.25 ± 0.29 ^AB^	0.60 ± 0.18 ^C^	0.72 ± 0.12 ^BC^
20:3n-6	3.71 ± 1.54 ^AB^	2.96 ± 0.84 ^AB^	4.31 ± 0.44 ^A^	2.31 ± 0.51 ^B^	2.65 ± 0.30 ^B^
20:4n-6	21.19 ± 4.59 ^BC^	17.76 ± 3.98 ^C^	19.01 ± 1.57 ^C^	27.75 ± 1.91 ^A^	25.49 ± 0.80 ^AB^
20:5n-3	1.11 ± 1.67 ^AB^	1.69 ± 0.42 ^A^	0.86 ± 0.15 ^AB^	0.29 ± 0.11 ^B^	0.30 ± 0.05 ^B^
22:2n-6	0.13 ± 0.10 ^AB^	0.05 ± 0.10 ^B^	0.00 ± 0.00 ^B^	0.27 ± 0.12 ^A^	0.03 ± 0.04 ^B^
22:4n-6	4.13 ± 0.18 ^A^	1.10 ± 0.52 ^C^	0.88 ± 0.22 ^C^	2.50 ± 0.99 ^B^	1.67 ± 0.34 ^BC^
22:5n-6	4.65 ± 1.80 ^C^	0.65 ± 0.23 ^B^	0.47 ± 0.13 ^B^	1.25 ± 0.48 ^B^	0.82 ± 0.18 ^B^
22:5n-3	1.45 ± 1.18 ^BC^	3.68 ± 1.06 ^A^	2.63 ± 0.45 ^AB^	1.08 ± 0.24 ^C^	1.21 ± 0.13 ^C^
22:6n-3	4.78 ± 1.70 ^C^	12.38 ± 4.10 ^A^	8.53 ± 1.38 ^B^	8.72 ± 0.65 ^B^	8.03 ± 0.59 ^BC^
Total PUFA	50.81	50.23	49.61	47.32	45.55

Within a row, values not sharing a letter are significantly different according to a one-way ANOVA (*p* < 0.05). Values are expressed as mean ± standard deviation (*n* = 6–11).

## Appendix A

**Table A1:** Composition of modified AIN-93 G rodent diets (research diets).

	10% Safflower ^a^ (D04092701)		3% Menhaden ^b^ (D04092703)		3% Flaxseed ^c^ (D04092711N)		10% Olive ^d^ (D16012101)		10% Lard ^e^ (D16012401)	
Macronutrient	g%	kcal%	g%	kcal%	g%	kcal%	g%	kcal%	g%	kcal%
Protein	21	20	21	20	21	20	21	20	21	20
Carbohydrate	60	58	60	58	60	58	60	58	60	58
Fat	10	22	10	22	10	22	10	22	10	22
Total		100		100		100		100		100
kcal/g	4		4		4		4		4	
Ingredient	g	kcal	g	kcal	g	kcal	g	kcal	g	kcal
Casein	200	800	200	800	200	800	200	800	200	800
L-Cystine	3	12	3	12	3	12	3	12	3	12
Corn starch	337	1347	337	1347	337	1347	337	1347	337	134
Maltodextrin 10	132	528	132	528	132	528	132	528	132	528
Sucrose	100	400	100	400	100	400	100	400	100	400
Cellulose, BW200	50	0	50	0	50	0	50	0	50	0
Safflower oil	97	873	68	611	68	611	0	0	0	0
Menhaden oil	0	0	29	262	0	0	0	0	0	0
Flax oil	0	0	0	0	29	262	0	0	0	0
Olive oil	0	0	0	0	0	0	97	873	0	0
Lard	0	0	0	0	0	0	0	0	97	873
t-Butylhydroquinone	0.02	0	0.02	0	0.02	0	0.02	0	0.02	0
Mineral Mix S10022	35	0	35	0	35	0	35	0	35	0
Vitamin mix V10037	10	40	10	40	10	40	10	40	10	40
Choline bitartrate	2.5	0	2.5	0	2.5	0	2.5	0	2.5	0
Total	966	4000	966	4000	966	4000	966	4000	966	4000

AIN-93G rodent diets (research diets catalogue number) modified to contain ^a^ 10% safflower oil, ^b^ 3% menhaden oil + 7% safflower oil, ^c^ 3% flax oil + 7% safflower oil, ^d^ 10% olive oil or ^e^ 10% lard.

## References

[1] American Cancer Society Breast Cancer Facts & Figures 2019–2020. https://www.cancer.org/content/dam/cancer-org/research/cancer-facts-and-statistics/breast-cancer-facts-and-figures/breast-cancer-facts-and-figures-2019-2020.pdf.

[2] Anand P., Kunnumakara A.B., Sundaram C., Harikumar K.B., Thakaran S.T., Lai O.K., Sung B., Aggarwal B.B. (2008). Cancer is a preventable disease that required major lifestyle changes. Pharm. Res..

[3] Parkin D.M., Boyd L., Walker L.C. (2011). 16. The fraction of cancer attributable to lifestyle and environmental factors in the UK in 2010. Br. J. Cancer.

[4] Gerber M. (2012). Omega-3 fatty acids and cancers: A systematic update review of epidemiological studies. Br. J. Nutr..

[5] Anderson B.M., Ma D.W. (2009). Are all n-3 polyunsaturated fatty acids created equal?. Lipids Health Dis..

[6] Liu J., Ma D.W.L. (2014). The role of n-3 polyunsaturated fatty acids in the prevention and treatment of breast cancer. Nutrients.

[7] Ziegler R.G., Hoover R.N., Pike M.C., Hildesheim A., Nomura A.M., West D.W., Wu-Williams A.H., Kolonel L.N., Horn-Ross P.L., Rosenthal J.F. (1993). Migration patterns and breast cancer risk in Asian-American women. J. Natl. Cancer Inst..

[8] Saadatian-Elahi M., Norat T., Goudable J., Riboli E. (2004). Biomarkers of dietary fatty acid intake and the risk of breast cancer: A meta-analysis. Int. J. Cancer.

[9] Simopoulos A.P. (2002). The importance of the ratio of omega-6/omega-3 essential fatty acids. Biomed. Pharmacother..

[10] Hilakivi-Clarke L., Clarke R., Onojafe I., Raygada M., Cho E., Lippman M. (1997). A maternal diet high in n-6 polyunsaturated fats alters mammary gland development, puberty onset, and breast cancer risk among female offspring. Nutrition.

[11] Liu J. (2015). “Plant- and Marine-derived N-3 Polyunsaturated Fatty Acids Prevent Mammary Tumor Development.” The Atrium, University of Guelph. https://atrium.lib.uoguelph.ca/xmlui/handle/10214/9126.

[12] Winikka L., Quach D., Harlow B., Brenner A., Munoz N., Tiziani S., de Graffenried L. (2018). Abstract P1-03-12: The ratio of omega-3 to omega-6 PUFAs impact cancer cell phenotype in the tumor microenvironment. Cancer Res..

[13] Dydjow-Bendek D., Zagoźdźon P. (2019). Total Dietary Fats, Fatty Acids, and Omega-3/Omega-6 Ratio as Risk Factors of Breast Cancer in the Polish Population—A Case-Control Study. In Vivo.

[14] Mourouti N., Panagiotakos D.B. (2016). The beneficial effect of a Mediterranean diet supplemented with extra virgin olive oil in the primary prevention of breast cancer among women at high cardiovascular risk in the PREDIMED Trial. Evid. Based Nurs..

[15] Menendez J.A., Papadimitropoulou A., Vellon L., Lupu R. (2006). A genomic explanation connecting “Mediterranean diet,” olive oil and cancer: Oleic acid, the main monounsaturated fatty acid of olive oil, induces formation of inhibitory “PEA3 transcription factor PEA3 DNA binding site” complexes at the Her-2/neu (erbB-2) oncogene promoter in breast, ovarian and stomach cancer cells. Eur. J. Cancer.

[16] Yang C.S., Landau J.M., Huang M.T., Newmark H.L. (2001). Inhibition of carcinogenesis by dietary polyphenolic compounds. Annu. Rev. Nutr..

[17] Van den Brandt P.A., Schulpen M. (2017). Mediterranean diet adherance and risk of postmenopausal breast cancer: Results of a cohort study and meta-analysis. Int. J. Cancer.

[18] Sieri S., Chiodini P., Agnoli C., Pala V., Berrino F., Trichopoulou A. (2014). Dietary fat intake and development of specific breast cancer subtypes. J. Natl. Cancer Inst..

[19] Turner L.B. (2011). A meta-analysis of fat intake, reproduction, and breast cancer risk: An evolutionary perspective. Am. J. Hum. Biol..

[20] Bassett J.K., Hodge A.M., English D.R., MacInnis R.J., Giles G.G. (2016). Plasma phospholipid fatty acids, dietary fatty acids, and breast cancer risk. Cancer Causes Control.

[21] Food and Agriculture Organization of the United Nations Fats and Fatty Acids in Human Nutrition: Report of an Expert Consultation. http://www.fao.org/3/a-i1953e.pdf.

[22] Xia H., Ma S., Wang S., Sun G. (2015). Meta-Analysis of Saturated Fatty Acid Intake and Breast Cancer Risk. Medicine.

[23] Liu J., Abdelmagid S.A., Pinelli C.J., Monk J.M., Liddle D.M., Hillyer L.M., Hucik B., Silva A., Subedi S., Wood G.A. (2018). Marine fish oil is more potent than plant-based n-3 polyunsaturated fatty acids in the prevention of mammary tumors. J. Nutr. Biochem..

[24] Salem N., Litman B., Kim H.Y., Gawrisch K. (2001). Mechanisms of action of docosahexanoic acid in the nervous system. Lipids.

[25] MacLennan M.B., Clarke S.E., Perez K., Wood G.A., Muller W.J., Kang J.X., Ma D.W. (2013). Mammary tumor development is directly inhibited by lifelong n-3 polyunsaturated fatty acids. J. Nutr. Biochem..

[26] Hamid R., Singh J., Reddy B.S., Cohen L.A. (1999). Inhibition by dietary menhaden oil of cyclooxygenase-1 and -2 in N-nitrosomethylurea-induced rat mammary tumors. Int. J. Oncol..

[27] Hardman W.E., Avula C.P., Fernandes G., Cameron I.L. (2001). Three percent dietary fish oil concentrate increased efficacy of doxorubicin against MDA-MB 231 breast cancer xenografts. Clin. Cancer Res..

[28] Hardman W.E. (2002). Omega-3 fatty acids to augment cancer therapy. J. Nutr..

[29] Rose D.P., Connolly J.M., Rayburn J., Coleman M. (1995). Influence of diets containing eicosapentaenoic or docosahexaenoic acid on growth and metastasis of breast cancer cells in nude mice. J. Natl. Cancer Inst..

[30] Rose D.P., Connolly J.M. (2000). Regulation of tumor angiogenesis by dietary fatty acids and eicosanoids. Nutr. Cancer.

[31] Senzaki H., Iwamoto S., Ogura E., Kiyozuka Y., Arita S., Kurebayashi J., Takada H., Hioki K., Tsubura A. (1998). Dietary effects of fatty acids on growth and metastasis of KPL-1 human breast cancer cells in vivo and in vitro. Anticancer Res..

[32] Li S., Zhou T., Li C., Dai Z., Che D., Yao Y., Li L., Ma J., Yang X., Gao G. (2014). High Metastaticgastric and breast cancer cells consume oleic acid in an AMPK dependent manner. PLoS ONE.

[33] Banim P.R., Luben R., Mctaggart A., Khaw K.-T., Wareham N., Hart A. (2011). Do Oleic Acid and N-3 Fatty Acids Prevent Pancreatic Cancer? Data from a UK Prospective Cohort Study Using 7-Day Food Diaries. Gastroenterology.

[34] Akl M.R., Ayoub N.M., Mohyeldin M.M., Busnena B.A., Foudah A.I., Liu Y.Y., Sayed K.A. (2014). Olive phenolics as c-Met inhibitors: (-)-Oleocanthal attenuates cell proliferation, invasiveness, and tumor growth in breast cancer models. PLoS ONE.

[35] Storniolo C.E., Martínez-Hovelman N., Martínez-Huélamo M., Lamuela-Raventos R.M., Moreno J.J. (2019). Extra Virgin Olive Oil Minor Compounds Modulate Mitogenic Action of Oleic Acid on Colon Cancer Cell Line. J. Agric. Food Chem..

[36] Liotti A., Cosimato V., Mirra P., Calì G., Conza D., Secondo A., Luongo G., Terracciano D., Formisano P., Beguinot F. (2018). Oleic acid promotes prostate cancer malignant phenotype via the G protein-coupled receptor FFA1/GPR40. J. Cell Physiol..

[37] Xiang F., Wu K., Liu Y., Shi L., Wang D., Li G., Tao K., Wang G. (2017). Omental adipocytes enhance the invasiveness of gastric cancer cells by oleic acid-induced activation of the PI3K-Akt signaling pathway. Int. J. Biochem. Cell Biol..

[38] Yang P., Su C., Luo X., Zeng H., Zhao L., Wei L., Zhang X., Varghese Z., Moorhead J.F., Chen Y. (2018). Dietary oleic acid-induced CD36 promotes cervical cancer cell growth and metastasis via up-regulation Src/ERK pathway. Cancer Lett..

[39] Coccia A., Mosca L., Puca R., Mangino G., Rossi A., Lendaro E. (2016). Extra-virgin olive oil phenols block cell cycle progression and modulate chemotherapeutic toxicity in bladder cancer cells. Oncol. Rep..

[40] Meng Y., Yuan C., Zhang J., Zhang F., Fu Q., Zhu X., Shu G., Wang L., Gao P., Xi Q. (2017). Stearic acid suppresses mammary gland development by inhibiting PI3K/Akt signaling pathway through GPR120 in pubertal mice. Biochem. Biophys. Res. Comm..

[41] Meng Y., Zhang J., Zhang F., Ai W., Zhu X., Shu G., Wang L., Gao P., Xi Q., Zhang Y. (2017). Lauric acid stimulates mammary gland development of pubertal mice through activation of GPR84 and PI3L/Akt Signaling Pathway. J. Agric. Food Chem..

[42] Binker-Cosen M.J., Richards D., Oliver B., Gaisano H.Y., Binker M.G., Cosen-Binker L.I. (2017). Palmitic acid increases invasiveness of pancreatic cancer cells AsPC-1 through TLR4/ROS/NF-κB/MMP-9 signaling pathway. Biochem. Biophys. Res. Commun..

[43] Wicha M.S., Lance A.L., Kidwell W.R. (1979). Effects of Free Fatty Acids on the Growth of Normal and Neoplastic Rat Mammary Epithelial Cells. Cancer Res..

[44] Dong J.Y., Zhang L., He K., Qin L.Q. (2011). Dairy consumption and risk of breast cancer: A meta-analysis of prospective cohort studies. Breast Cancer Res. Treat..

[45] Guo J., Wei W., Zhan L. (2015). Red and processed meat intake and risk of breast cancer: A meta-analysis of prospective studies. Breast Cancer Res. Treat..

[46] Lingwood D., Simons K. (2010). Lipid rafts as a membrane-organizing principle. Science.

[47] Patra S.K. (2008). Dissecting lipid raft facilitated cell signaling pathways in cancer. Biochem. Biophys. Acta.

[48] Lee E.J., Yun U.J., Koo K.H., Sung J.Y., Shim J., Ye S.K., Hong K.M., Kim Y.N. (2014). Down-regulation of lipid raft-associated onco-proteins via cholesterol-dependent lipid raft internalization in docosahexaenoic acid-induced apoptosis. Biochim. Biophys. Acta.

[49] Burdge G.C., Calder P.C. (2005). Conversion of α-linolenic acid to longer-chain polyunsaturated fatty acids in human adults. Reprod. Nutr. Dev..

[50] Benoit V., Relic B., Leval X., Chariot A., Merville M.P., Bours V. (2004). Regulation of HER-2 oncogene expression by cyclooxygenase-2 and prostaglandin E2. Oncogene.

[51] Larsson S.C., Kumlin M., Ingelman-Sundberg M., Wolk A. (2004). Dietary long-chain n-3 fatty acids for the prevention of cancer: A review of potential mechanisms. Am. J. Clin. Nutr..

[52] Wang D.D., Hu F.B. (2017). Dietary Fat and Risk of Cardiovascular Disease: Recent Controversies and Advances. Annu. Rev. Nutr..

[53] Burns J.L., Nakamura M.T., Ma D.W. (2018). Differentiating the biological effects of linoleic acid from arachidonic acid in health and disease. Prostaglandins Leukot. Essent. Fat. Acids.

[54] Li Q., Wang M., Tan L., Wang C., Ma J., Li N., Li Y., Xu G., Li J. (2005). Docosahexaenoic acid changes lipid composition and interleukin-2 receptor signaling in membrane rafts. J. Lipid Res..

[55] Ravacci G.R., Brentani M.M., Tortelli T., Torrinhas R.S., Saldanha T., Torres E.A., Waitzberg D.L. (2013). Lipid raft disruption by docosahexaenoic acid induces apoptosis in transformed human mammary luminal epithelial cells harboring her-2 overexpression. J. Nutr. Biochem..

[56] Muller W.J., Sinn E., Pattengale P.K., Wallace R., Leder P. (1988). Single-step induction of mammary adenocarcinoma in transgenic mice bearing the activated c-neu oncogene. Cell.

[57] Statistics Canada Table 13-10-0769-01 Percentage of Total Energy Intake from Fat, by Dietary Reference Intake Age-Sex Group, Household Population Aged 1 and over, Canadian Community Health Survey (CCHS)—Nutrition, Canada and Provinces.

[58] Harris W.S., Mozaffarian D., Rimm E., Kris-Etherton P., Rudel L.L., Appel L.J., Engler M.M., Engler M.B., Sacks F. (2009). Omega-6 fatty acids and risk for cardiovascular disease: A science advisory from the American Heart Association Nutrition Subcommittee of the Council on Nutrition, Physical Activity, and Metabolism; Council on Cardiovascular Nursing; and Council on Epidemiology and Prevention. Circulation.

[59] Kim W., McMurray D.N., Chapkin R.S. (2010). n-3 polyunsaturated fatty acids—Physiological relevance of dose. Prostaglandins Leukot. Essent. Fat. Acids.

[60] U.S. Departments of Health and Human Services (HHS) and Agriculture (USDA), Dietary Guidelines Advisory Committee Scientific Report of the 2015 Dietary Guidelines Advisory Committee. http://www.health.gov/dietaryguidelines/2015-scientific-report/.

[61] Agence Française de Sécurité Sanitaire des Aliments (AFSSA) (2010). Avis de l’Agence française de sécurité sanitaire des aliments relatif à l’actualisation des apports nutritionnels conseillés pour les acides gras (Opinion of the French Food Safety Agency on the Update of French Population Reference Intakes (RDIs) for Fatty Acids). Request No. 2006-SA-0359. https://www.anses.fr/fr/content/avis-de-l%E2%80%99agence-fran%C3%A7aise-de-s%C3%A9curit%C3%A9-sanitaire-des-aliments-relatif-%C3%A0-l%E2%80%99actualisation-de-2.

[62] Barlow L.J., Zhang J.-T. (2017). Abstract 4415: Fatty acid synthase-mediated palmitate production impacts epidermal growth factor receptor signaling to regulate specificity protein 1 in breast cancer cells. Mol. Cell Biol. Genet..

[63] Holder A.M., Gonzalez-Angulo A.M., Chen H., Akcakanat A., Anh-Do K., Symmans F., Pusztai L., Hortobagyi G., Mills G.B., Meric-Bernstam F. (2012). Abstract 682: Increased stearoyl-CoA desaturase 1 expression is associated with shorter survival in breast cancer patients. Clin. Res..

